# Supplementary material for: Global genomic population structure of wild and cultivated oat reveals signatures of chromosome rearrangements
Source: Nat Commun. 2025 Oct 29;16:9486. doi: 10.1038/s41467-025-57895-3 (PMC12572283; doi:10.1038/s41467-025-57895-3)
Supplement: Supplementary file 1 — Supplementary Information [file 41467_2025_57895_MOESM1_ESM.pdf]

Global genomic population structure of wild and cultivated oat reveals signatures of chromosome rearrangements

Bekele *et al.*

Supplementary Note 1. Species reclassification

In this study we have preserved the species identifier that was assigned by the original collector or genebank. In some cases, these names may be erroneous, or they may be affected by an evolving consensus on oat taxonomy. The project metadata in Supplementary Data 1 contains a field for potential taxonomic revisions. Currently we have suggested the reclassification of 27 accessions to *A. byzantina* based on their membership in this genetically distinct group. We also found 11 accessions identified as *A. sterilis* that were classified in populations belonging mostly to *A. sativa*. This includes one (CN_23397) in P06, six (TL.S325, TL.S313, TL.S300, TL.S277, NAM_CAV1627, CN_20562) in P11, two (CN_20165 and CN_20161) in P12, and two (TL.S289 and CN_22599) in P21. Most of these populations contain winter oat. We have checked the phenotype of many of these accessions, and they appear to have *A. sterilis* seed types, but we cannot rule out that they are not feral hybrids with *A. sativa* (sometimes classified as *A. hybrida*). Further investigation of these lines may shed light on the dynamics of gene flow. In future updates of the metadata (Supplementary Data 1) on the GrainGenes archive, this field could be used for other species revisions or comments related to classification. For example, the species name *A. ludoviciana*[1] could be used to describe many accessions currently classified as *A. sterilis*[2] and a future botanical revision could inform us that this classification is consistent with one of the current *A. sterilis* populations. Our study included four accessions of *A. ludoviciana*. Two of these did not have adequate data to assign them to a population, but the other two were both assigned to P21. *Ludoviciana* appears to be a winter habit form of sterilis, which may not have been recognized as a separate category by genebanks or collectors (possibly because of the difficulty in measuring vernalization requirement), and it is possible that other species classifies as *A. sterilis* could fall into this category.

Supplementary Note 2. Potential for experimental bias

While these experiments followed similar protocols, all GBS experiments contain slight incidental differences that affect fragment size selection, sequencing depth, and variation among samples. Because each experiment focused on different sets of germplasm, we cannot rule out the possibility that some observed population structure is affected by experimental bias. Nevertheless, most results are consistent with previous analyses and with prior biological knowledge of the genus. Furthermore, while some populations are exclusive to specific experiments, most populations contain taxa from many experiments, indicating that population structure is generally independent of experimental bias. For example, while P09 originates

almost exclusively from the CORE-Spring, POGI and Cornell experiments, the same three experiments contributed to most other populations of *A. sativa*.

Supplementary Discussion 1. Duplicated accessions in the global oat diversity panel

Duplicated accessions are a common and complex issue in genebanks and biodiversity analyses[3]. We know that many genebank accessions are heterogeneous because collectors often sampled multiple plants as one accession, or single plants may have been heterozygous. Accessions also become mixed or contaminated, especially when they are grown out for multiplication. Likewise, we know that many landraces (and even cultivated varieties) contain heterogeneity. Unfortunately, heterogeneity can also arise through many types of error. It is common practice (as followed in these experiments) to sample a single seed to represent an accession. Ideally, we would perform separate analyses on multiple seeds from every accession, but at what cost? One seed from each of 9000 accessions provides much more information than nine seeds from each of 1000 accessions.

To explore duplication and heterogeneity of genebank accessions, we included a small internal experiment, in which three or four seeds were sampled from each of 99 genebank accessions (Table 1). We also included an “accidental” experiment, as some varieties and landraces were sampled multiple times due to the merging of different experiments that were planned and conducted independently by different investigators. In the latter case, an effort was required to cross-reference entries where names were identical or contained enough similarity that they could be assumed to be from the same named variety.

A summary was developed (Supplementary Data 3) containing 747 duplicated (or potentially duplicated) accessions among 1837 unique GBSIDs. An analysis of genomic distance among duplicated taxa (Supplementary Fig. 1) compares the average distance between pairs of subsamples from each of 99 genebank accessions *vs.* the average distance between pairs of samples from similarly-named accessions. The different levels of heterogeneity among genebank accessions suggests that many accessions contain substantial heterogeneity, which could be investigated further.

Surprisingly, the level of heterogeneity among named varieties was of the same frequency and magnitude as that of the genebank accessions. These differences are likely due to a variety of

reasons including heterogeneous accessions, different seed sources, and mislabeling. Where duplicates exist, additional data mining is recommended prior to targeted analysis such as genome sequencing. For example, we note that the oat cultivar ‘Firth’ is duplicated in 6 experiments. Of the 15 possible pairwise genetic distances, the five involving Firth from the Cornell experiment (Table 1) gave an average distance of 0.21 from other Firth accessions, while the remaining distances had an average pairwise distance of 0.002. Thus, we can infer that the Firth sampled in the Cornell experiment is probably from a misidentified seed source. In contrast, nine taxa represent an historical founding landrace called ‘Red Algerian’, all with small-to- moderate genetic distances. Because this is an old landrace with no definitive origin, most of these differences likely represent heterogeneity among seed sources rather than experimental error.

While duplication introduces a small amount of uncontrolled bias, this bias is assumed to be random. No duplicates were removed from this study because the genetic similarity between duplicates followed a gradient of certainty with no obvious cutoff, and because heterogeneity among duplicates represents real data that would otherwise be lost.

Supplementary Discussion 2. Robustness of SNP-based diversity analysis

In this study, we employed a factorial analysis by MDS to provide a visualization of global diversity within hexaploid *Avena* species or within cultivated *A. sativa*. This visualization was conducted in a large SNP-based data matrix (Matrix50) having some taxa with substantial missing data. This analysis was then interpreted using a population-based analysis based on a more complete data matrix (Matrix80), in which taxa having more than 10% missing data were omitted. Thus, some taxa that appear in the MDS plots are not fully characterized by their population membership. However, it may be possible to make inferences about population membership of taxa with missing data based on genetic proximity to other taxa. In this supplement, we examine the robustness of the factorial-based visualizations, as well as the consistency of SNP calls based on two additional SNP calling methods.

Exploratory analysis by both PCA and MDS were performed using the full data set (Matrix50) as well as the filtered set (Matrix80). In addition, MDS was performed for data from the two alternate SNP calling methods (Supplementary Methods 1 and 2). The MDS *vs.* PCA plots in the full data set (Supplementary Fig. 2a *vs.* 2b) showed a different shape and appearance. By examining taxa with the highest proportion of missing data, we determined that these lines were

highly skewed toward the origin in the PCA plot, but not in the MDS plot. Most strikingly, many lines from the Jordanian experiment (circled in red) had very high proportions of missing data. In the PCA plot (Fig. 2b) these lines were oriented toward the origin relative to other lines, whereas in the MDS plot (Fig. 2a) these lines clustered together with taxa in three different regions of the MDS. In contrast, the MDS *vs.* PCA plots in the more complete, highly filtered data set (Supplementary Fig. 2c *vs.* 2d) were much more similar in structure.

The bias of PCA analysis with missing data is a well-known phenomenon, but our goals were (1) to determine if the MDS and PCA plots showed similar structure in the more complete Matrix80 data (they do) and (2) whether the MDS plot provided a better representation of lines having missing data. In the second case, we can say that the distribution of lines with missing data is more logical in the MDS plot than in the PCA plot, but we still recommend cautious interpretation of the MDS positions for lines with missing data.

We also evaluated whether the use of the Sang (*A. sativa*) reference genome affected the proportion of SNPs called in non-sativa species. Because of the diversity in read depth among different experiments, we filtered only those taxa having read counts between 1E6 and 3E6. This gave a similar average number of reads for taxa in each of the three major species, as summarized in Supplementary Table 2. Among these three species, *A. byzantina* had the highest proportion of missing data while *A. sterilis* had the lowest. From this we conclude that the proportion of missing data (i.e. un- called SNPs) is more dependent on other factors (e.g. read quality, sequencing technology) than on how closely a genome is related to the Sang reference genome.

In Supplementary Figs. 2e and 2f, the full sets of taxa based on two alternate methods of SNP calling are shown using MDS. While there appear to be subtle differences between these analyses, the overall shape of the MDS plot is very similar among all three data sets. Additionally, we conducted a correlation analysis and Mantel test to compare the complete distance matrices between the SNPs called using the Sang reference (Fig. 2a) *vs.* those called using the GS7 reference (Fig. 2f). Raw data used for the Mantel test is downloadable from the GrainGenes project site (https://wheat.pw.usda.gov/GG3/content/global-oat-genomic-diversity-project). The Pearson correlation coefficient between these sets of distances was very high (r=0.9935) with a significance of P<0.001 based on 999 permutations.

Supplementary Discussion 3. Projection of reference genomes on global oat diversity

One of the objectives of this study was to identify diverse oat accessions for the purpose of developing a hexaploid oat pan genome[4]. When it was initiated, the oat pan genome study used reference genomes that were selected in part based on an earlier version of the current data set. That earlier data set did not include many of the taxa that were added subsequently. In particular, we later added a much larger set of *A. sterilis* taxa from the BioMob project, as well as most of the Australian taxa (Table 1). We then wished to identify how well this expanded hexaploid oat diversity space was represented by the accessions from oat pan genome, and to identify any gaps that might be filled in subsequent projects (see Supplementary Methods 3). The MDS plot from the full data set resolved the positions of six reference genomes (Supplementary Fig. 3a) while the reduced analysis allowed better resolution of the remaining reference genomes (Supplementary Fig. 3b). Supplementary Table 3 shows the closest GBS taxa to each reference genome based on data from the Sang SNPs *vs.* data from the GS7 SNPs, as well as the population membership of the nearest genome based on either method. From Fig. 3a and the Sang projections in Supplementary Table 3, it was apparent that reference genomes A-byzantina, TN4, CN25955, TN1, and FM13 fell within populations P01, P03, P04, P05 and P06, respectively, with no reference genome representing P02. From Fig. 3b and Table 3, the remaining reference genomes covered populations P07, P09, P11-P17, P20, and P21. Population membership based on the GS7 SNPs was different for four accessions (bold in Table 3), but both methods identified the same five populations without representation, including P02, P08, P10, P18, and P19.

From this we infer that conclusions about highly similar lines in adjacent populations are subject to some variability. Nevertheless, these results demonstrate that the 29 hexaploid reference taxa from the pan genome project provide a good coverage of most of the hexaploid *Avena* diversity space.

The most notable exception is population P02. Thus, sequencing of one or more lines from population P02 should be prioritized in future efforts in order to confirm or refine our hypothesis that P02 is the progenitor of cultivated A. *byzantina.* Another notable exception is population P08, which contains the southern USA lines. Although the Australian lines (P07) are genetically similar to P08, we had assumed that the line Victoria would cover the Southern USA types, which it did not. Thus, this population should also be prioritized in future

sequencing efforts.

Supplementary Method 1. SNPs called using the GS7 Reference genome

A supplementary analysis was carried out using SNPs called based on the GS7 reference genome, reported in the oat pan genome study[4]. This analysis was conducted after the reference genomes in the oat pangenome were assembled, when it was determined that the accession GS7 had one of the highest quality assemblies. Thus, we intended to compare our SNP-calling methods based on the Sang reference genome[5] (assembled by short reads) to independently called SNPs based on the GS7 reference genome. Methods for GS7-based SNP calling and filtering are described in the companion study[4]. The use of these different methods provided an opportunity to confirm that results were not affected by bioinformatics methodology.

Supplementary Method 2. Haplotag analysis

A supplementary analysis was carried out using the software "Haplotag"[6] for reference-free SNP calling. The purpose of this method was to compare results reported in the main manuscript to results that would be obtained with Haplotag, since Haplotag has been used for many previous GBS reports by the oat genomics research community. In this method, we first filtered the pre-discovered loci and tag-level haplotypes described by Bekele *et al*.[7] to include only those loci where one and only-one tag-level haplotype matched perfectly to the Sang genome[5], producing the list of physically-located 64-base loci described by Tinker *et al.* [8]. We then aligned all novel tags from the master tag-count file (produced by UNEAK software[9] directly from the raw fastq read data) to the tag-level haplotype list, allowing for only one additional SNP beyond the previously-known SNP locations within each 64-base locus. We further eliminated all loci that did not provide a unique match with a novel tag-level haplotype. The resulting rebuilt 'HTLoci' and 'HTalleles' files were then used in the standard Haplotag production pipeline without further modification.

Supplementary Method 3. Projection of SNP calls onto reference genomes

Genome positions of the SNP loci from the Matrix50 data set were identified within the pseudomolecules of the Sang genome. For each locus, a 400bp DNA fragment was extracted beginning 10bp upstream from a given SNP using the software SeqKit[10]. The extracted Sang sequences were formatted as a fasta query file. The remainder of the reference genomes were formatted as searchable NCBI BLAST+[11] databases. Each genome was then searched using the

Sang query with BLAST parameters *"-evalue 1e-180 -max_target_seqs 1 -max_hsps 1 -outfmt 0*". The resulting output files were parsed using a small Pascal script which identified the nucleotide matching the SNP (at the 10th base pair) in the best matching HSP for each locus. Loci were scored as ambiguous (N) if no match was found. The resulting SNP calls for each of 29 hexaploid reference genomes[4] were merged with the GBS data in the Martix50 data file. An MDS analysis was performed using TASSEL[12] and visualized using DARWin software[13] The associated distance matrix was parsed to identify the closest GBS-based taxa having a population-based assignment to each reference genome. A reduced data set containing only the lines from *A. sativa* populations was analyzed by the same methods. Similar methods were conducted to project SNPs using the GS7 SNP calls (see Supplementary Discussion 2) as reported in our companion paper[4].

Supplementary Table 1. Details of sequencing for experiments contributing to global genomic diversity of oat.

Project	Tissue collection place	Seq. centre	Seq. device
Aberystwyth	IBERS, UK	IPK, Germany	Illumina NovaSeq 6000
Ancestors	AAFC, Ottawa, Canada	IBERS, UK	Illumina Hiseq 2000
Australia	University of Adelaide - Waite Campus	University of Minnesota, USA	Illumina Hiseq 2500
BioMob	AAFC Saskatoon, Canada	NRC Saskatoon, Canada	Illumina NovaSeq 6000
China	Triticeae Research Institute, Sichuan, China	AAFC Ottawa, Canada	Illumina NextSeq 500
CORE_AFRI	USDA Aberdeen, USA	NRC Saskatoon, Canada	Illumina Hiseq 2000
CORE_Spring	USDA Aberdeen, USA	NRC Saskatoon, Canada	Illumina Hiseq 2000
CORE_Winter	USDA Aberdeen, USA	NRC Saskatoon, Canada	Illumina Hiseq 2000
Cornell	Cornell University, USA	University of Minnesota, USA	Illumina Hiseq 2500
Fusarium	CBGP, UPM-INIA, Spain	Genome Quebec, Canada	Illumina Hiseq 2500
IOI	AAFC, Ottawa, Canada	NRC Saskatoon, Canada	Illumina Hiseq 2000
Jordan	AAFC Saskatoon, Canada	AAFC Saskatoon, Canada	Illumina MiSeq
KLAR	KLAR, Germany	Genome Quebec, Canada	Illumina Hiseq 2500
Mediterranean	IAS, CSIC, Spain	Genome Quebec, Canada	Illumina Hiseq 2500
NAM	IBERS, UK	Genome Quebec, Canada	Illumina Hiseq 2500
NSGC	USDA Aberdeen, USA	USDA Aberdeen, USA	Illumina Novaseq6000
POGI	USDA Fargo, ND, USA	USDA Fargo, ND, USA	Illumina NextSeq 500

Supplementary Table 2. Proportion of missing data in taxa from three species.

Species	Avg. N. reads	Avg. proportion missing	Count of taxa meeting criterion
A. byzantina	2,088,347	0.48	260
A. sativa	2,018,485	0.45	3193
A. sterilis	2,094,306	0.43	443

Supplementary Table 3. Population membership of reference genomes.

**Based on Sang SNP calls**				**Based on GS7 SNP calls**		
**reference**	**Closest GBS line**	**Distance**	**Population (*K*=21)**	**Closest GBS line**	**Distance**	**Population (*K*=21)**
A. byzantina	PI 258586	0.0312	P01	PI 258586	0.0365	P01
**Amagalon**	**FABRSE**	**0.1G01**	**P04**	**CN 25974**	**0.1975**	**P11**
Aslak	Aslak	0.0013	P15	Aslak	0.0024	P15
Bannister	Bannister	0.0289	P07	Bannister	0.0326	P07
Bilby	Bilby	0.0194	P07	Bilby	0.0225	P07
**Bingo**	**Sang**	**0.0502**	**P17**	**DCAs10**	**0.0519**	**P15**
Clintland60	MORTON	0.0405	P20	MORTON	0.0440	P20
CN25955	CN 25956	0.0908	P04	CN 25956	0.0920	P04
Delfin	Delfin	0.0027	P17	Delfin	0.0045	P17
FM13	FM13	0.0174	P06	FM13	0.0188	P06
**Gehl**	**ND160**	**0.0048**	**P18**	**Gehl**	**0.0083**	**P11**
**GMI423**	**BGMN318**	**0.0575**	**P15**	**Triple Crown**	**0.0648**	**P11**
GS7	IL07-8721	0.0643	P14	IL07-8721	0.0659	P14
Hatives	PI 573559	0.0162	P11	PI 573559	0.0192	P11
HiFi	HiFi	0.0053	P09	HiFi	0.0083	P09
Leggett	SA130278	0.0038	P16	Leggett	0.0066	P16
Lion	NORD 15/325	0.004	P17	NORD 15/325	0.0048	P17
Morgan	AC Morgan	0.0022	P17	AC Morgan	0.0050	P17
Nicolas	OA1331-5-0-21	0.0036	P16	OA1331-5-0-21	0.0074	P16
OT3098	SA130460	0.0037	P16	SA130460	0.0069	P16
OT380	OT380	0.0037	P11	OT380	0.0072	P11
Park	CN 42524	0.0066	P13	CN 42524	0.0081	P13
PI182478	Bullion	0.006	P12	Bullion	0.0092	P12
Rhapsody	Tardis	0.027	P21	Tardis	0.0304	P21
Sang	Sang	0.0001	P11	Sang	0.0003	P11
TN1	ST0TN1	0.0099	P05	ST0TN1	0.0135	P05
TN4	CN 20957	0.0965	P03	CN 20957	0.0995	P03
Victoria	Victoria	0.0118	P21	Victoria	0.0149	P21
Williams	Williams	0.0092	P07	Aus_195	0.0124	P07

Reference genomes highlighted in bold differ slightly in assigned population between SNP calling methods.

a

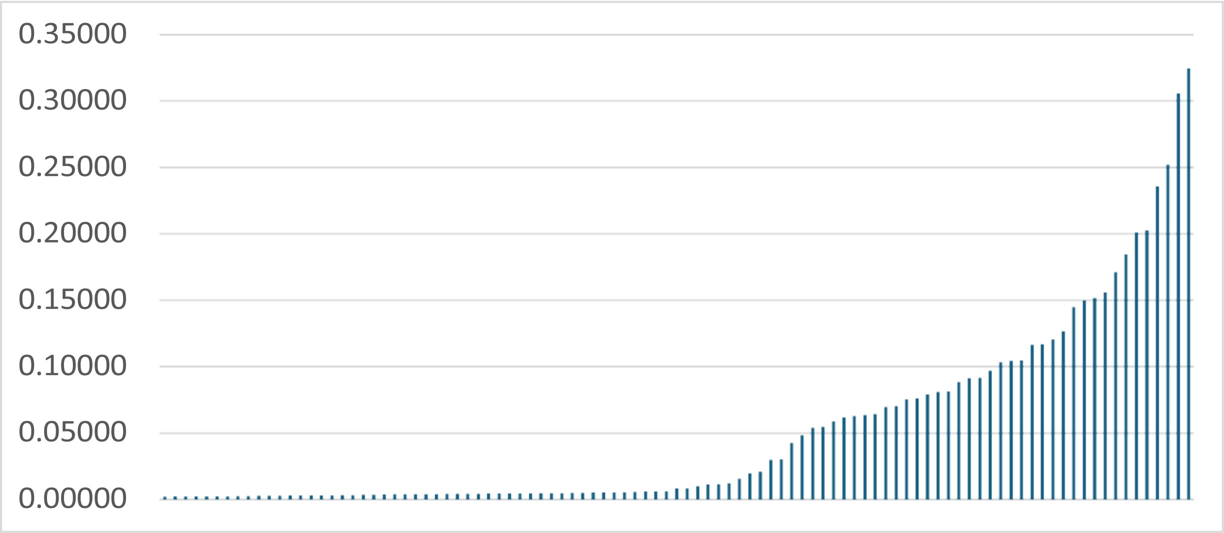

b

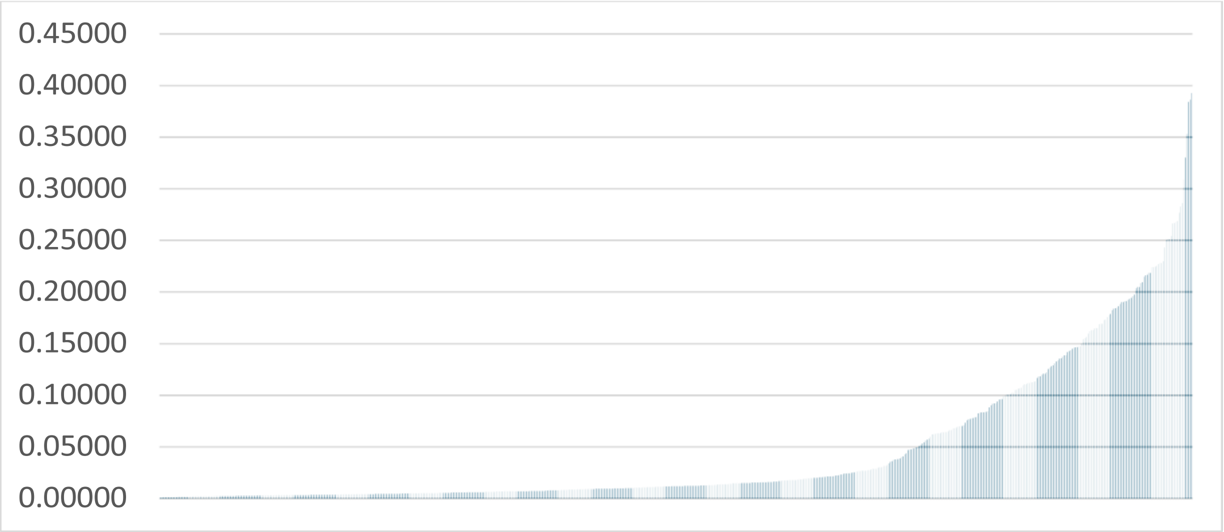

Supplementary Fig. 1. Analysis of Duplicated taxa. Average genetic distance among pairs of identically named accessions generated from pairs of potential duplicates, as shown in Supplementary Data 3. Each vertical bar represents the average genetic distance among all possible pairs within a single potentially duplicated accession, sorted left to right by increasing average distance (*i.e.*, there is no scale on this axis). **(a)** 99 accessions sampled three to four times each from the Canadian genebank. **(b)** 648 named accessions duplicated across different experiments, not necessarily from the same seed source. Source data are provided as a Source Data file.

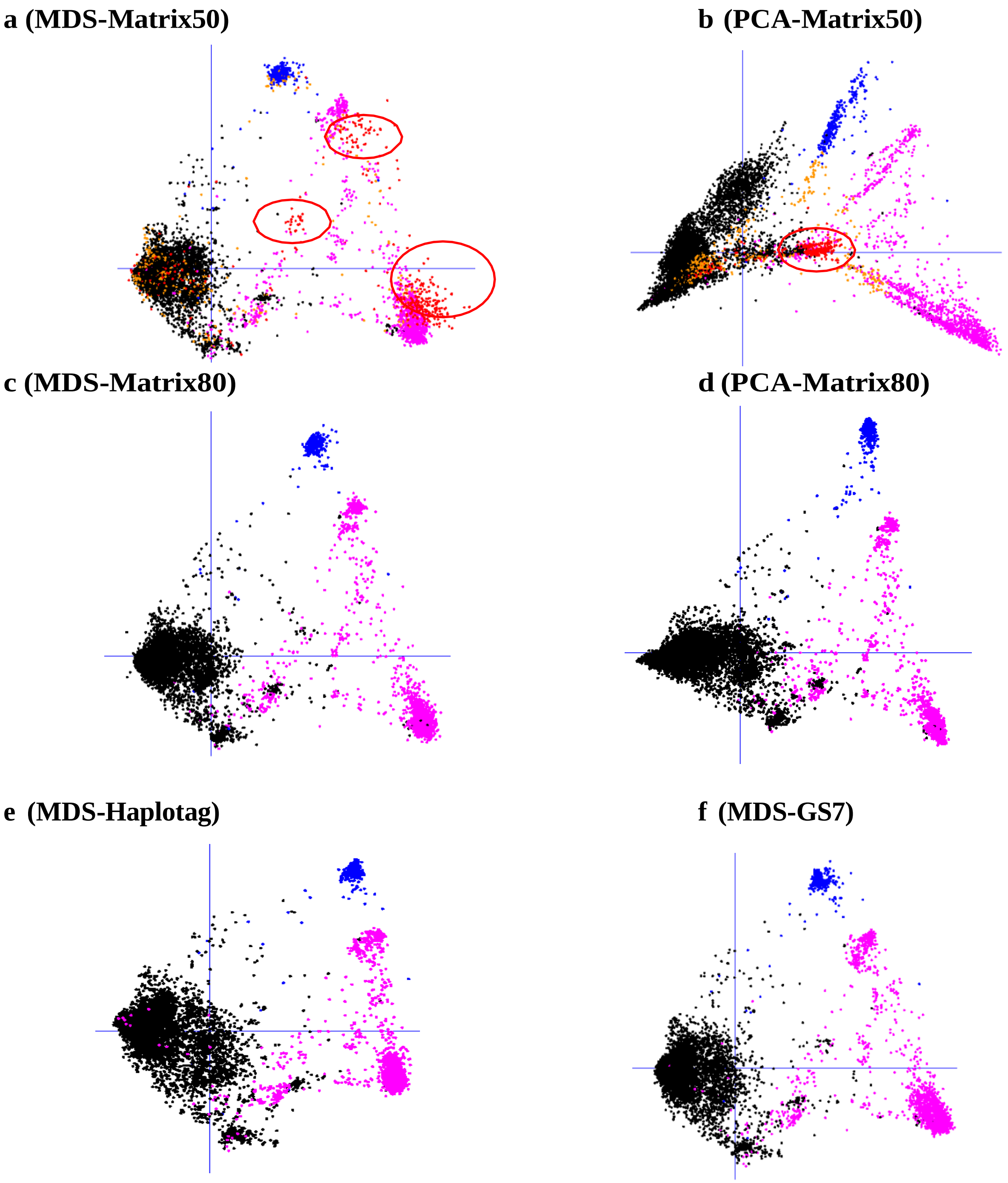

Supplementary Fig. 2. Robustness of SNP calls and visualization methods. (a and **b)** analysis of the full SNP data set from Matrix50 by MDS **(a)** *vs*. PCA **(b).** Points with missing genotype data are coloured red (80-100%) or orange (60-80%). **(c** and **d)** analysis of filtered data set with imputed genotypes (Matrix80) by MDS *vs*. PCA, respectively. **(e)** analysis of SNPs called using Haplotag software by MDS. **(f)** analysis of SNPs called using the GS7 reference genome by MDS. In all plots, points that represent the *A. byzantina* cluster are coloured blue, while those representing *A. sterilis* lines are magenta. The axes of all plots are mirrored oriented to orient the *A. byzantina* cluster in the top right quadrant. Axis scales are not reported because they are arbitrary in the MDS analyses, and we are only interested in the shape of these plots for descriptive purposes. Source data are provided as a Source Data file.

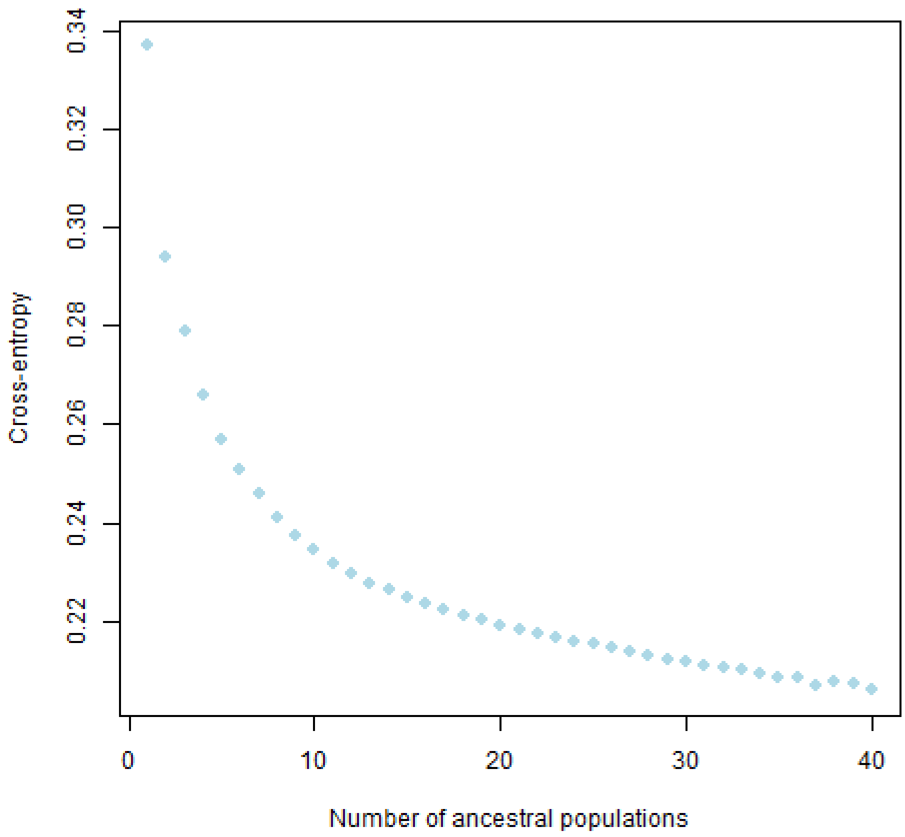

Supplementary Fig. 3. Cross-entropy *vs.* cluster size. Cross-entropy *vs.* cluster size (K) based on five sNMF iterations per cluster (K) = 1 – 40. Source data are provided as a Source Data file.

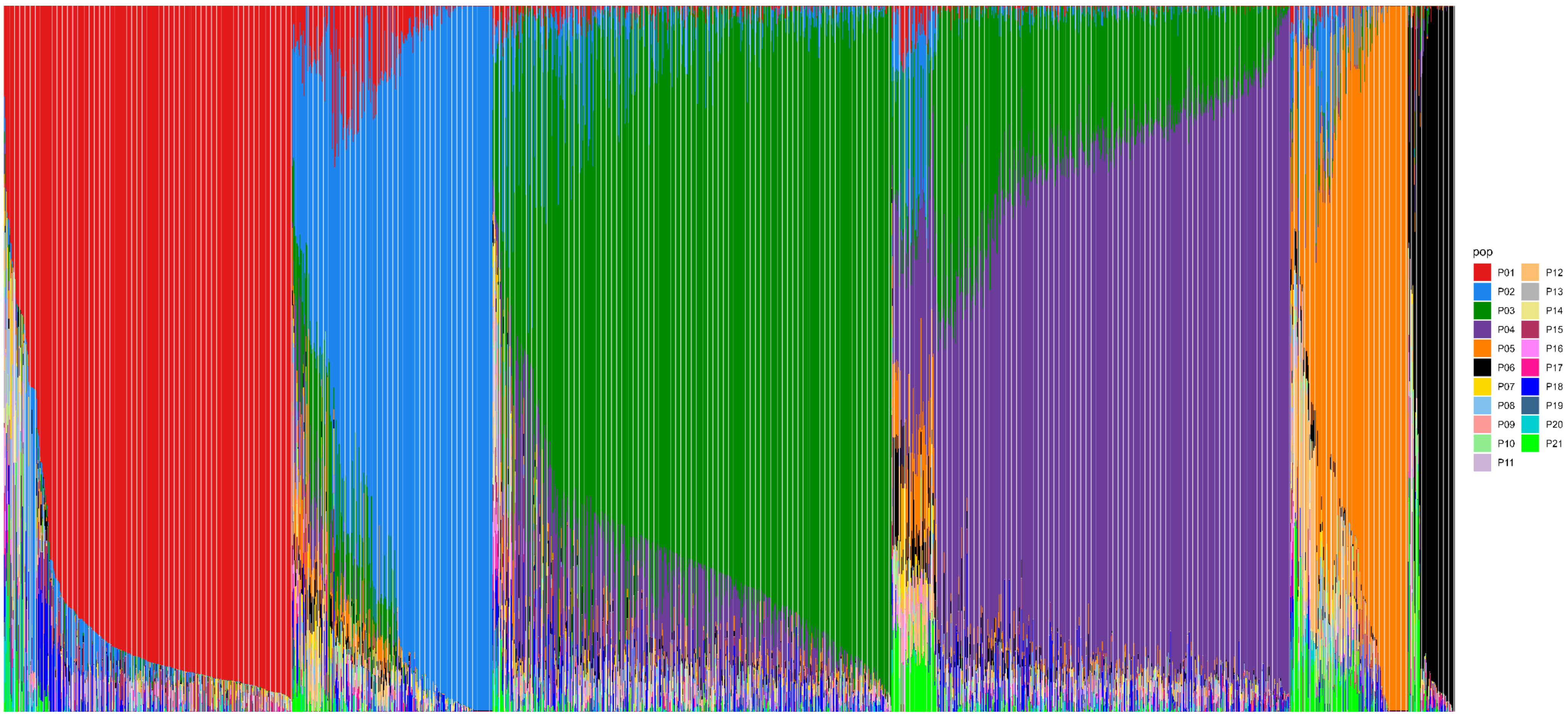

Supplementary Fig. 4. Admixture plot – selected populations. Admixture plot from *K*=21 showing taxa assigned to populations P01, P02, P03, P04, P05, and P06. Source data are provided as a Source Data file.

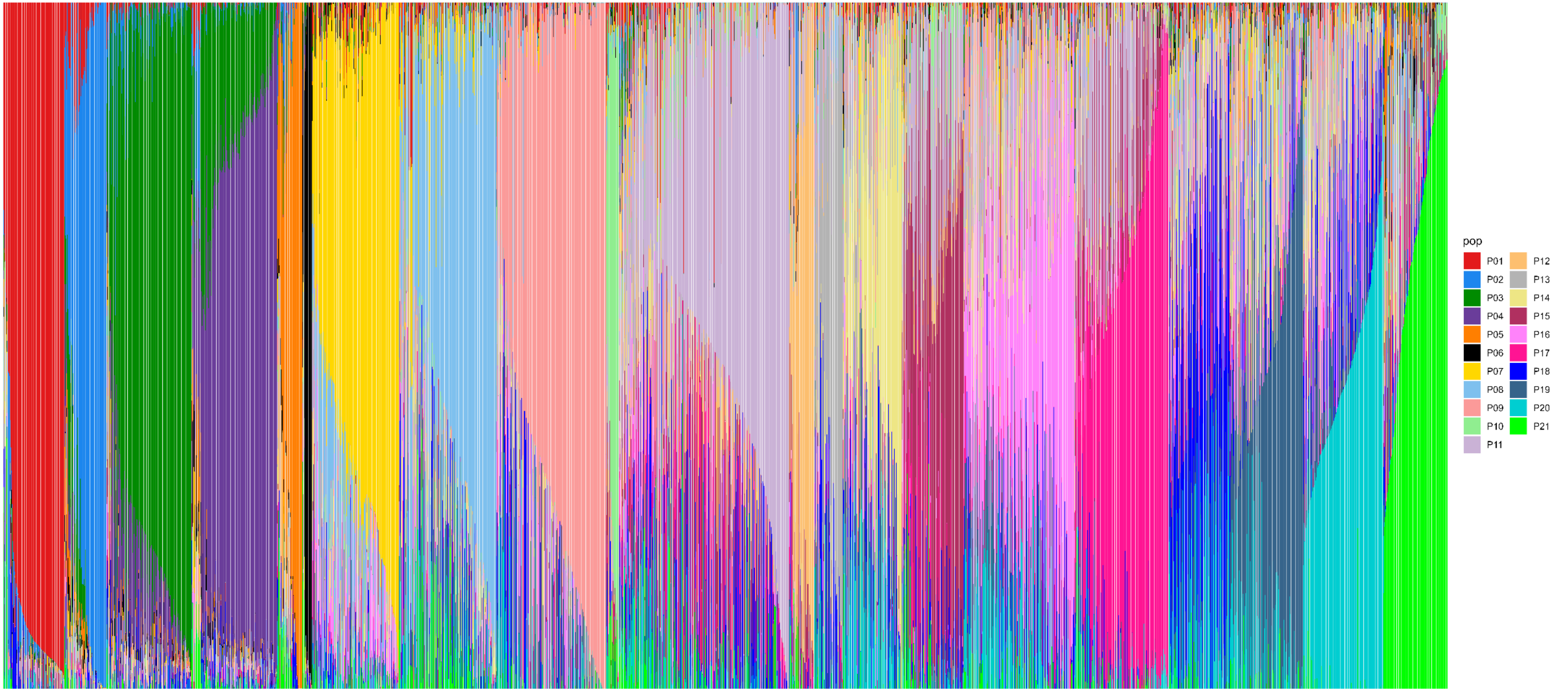

Supplementary Fig. 5. Admixture plot – all populations. Admixture plot from *K*=21 showing taxa assigned to all populations. Source data are provided as a Source Data file.

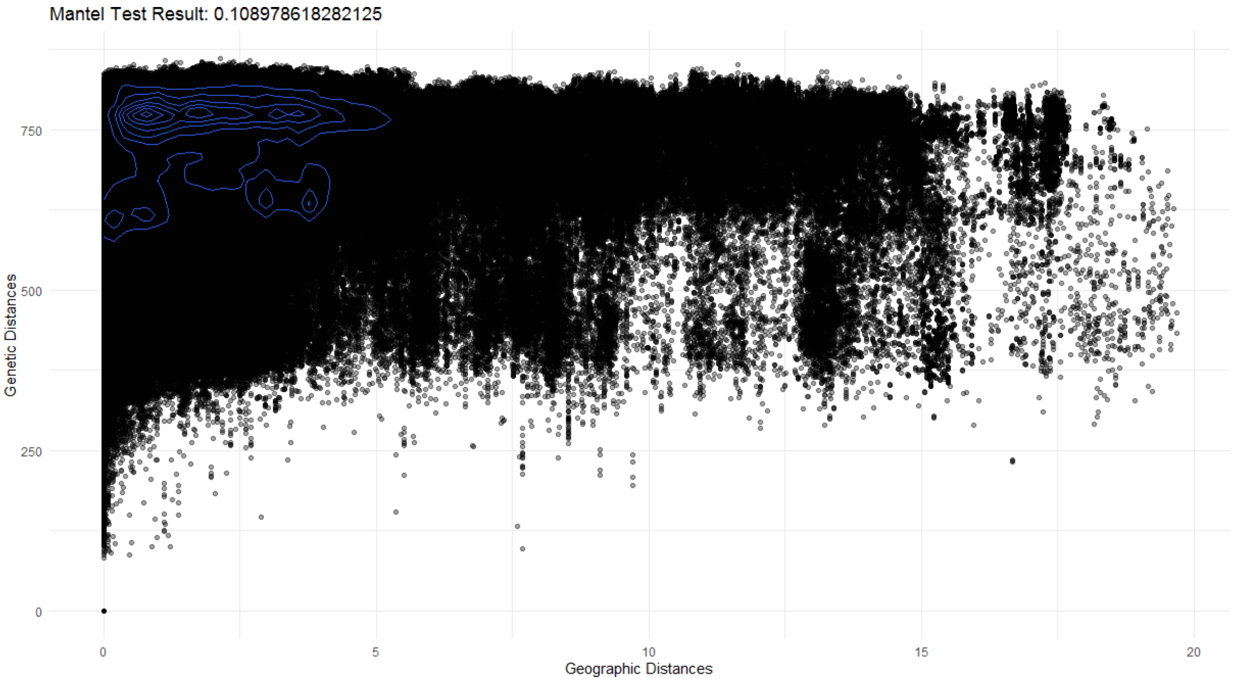

Supplementary Fig. 6. Pairwise genetic distance *vs.* geographical distance. Pairwise genetic distance *vs*. Geographical (million meters) distance between collection sites of 2279 accessions. Raw data used for the Mantel test is downloadable from the GrainGenes project site (https://wheat.pw.usda.gov/GG3/content/global-oat-genomic-diversity-project). Source data are provided as a Source Data file.

a

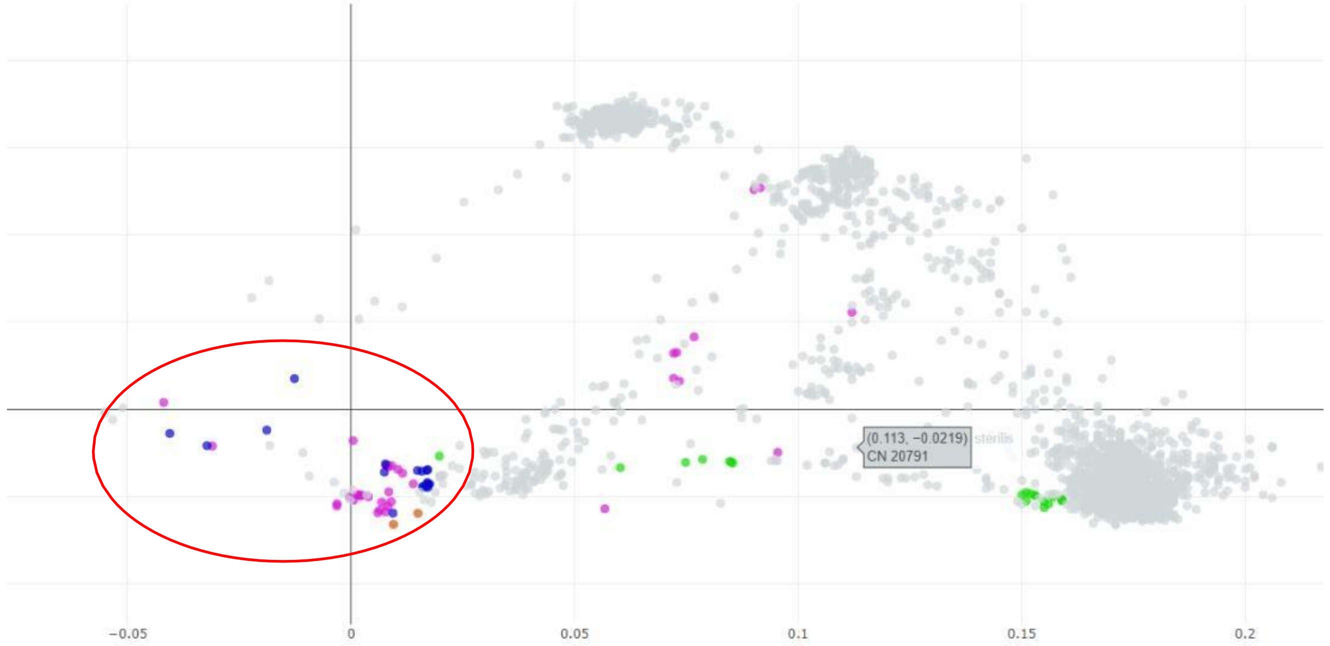

b

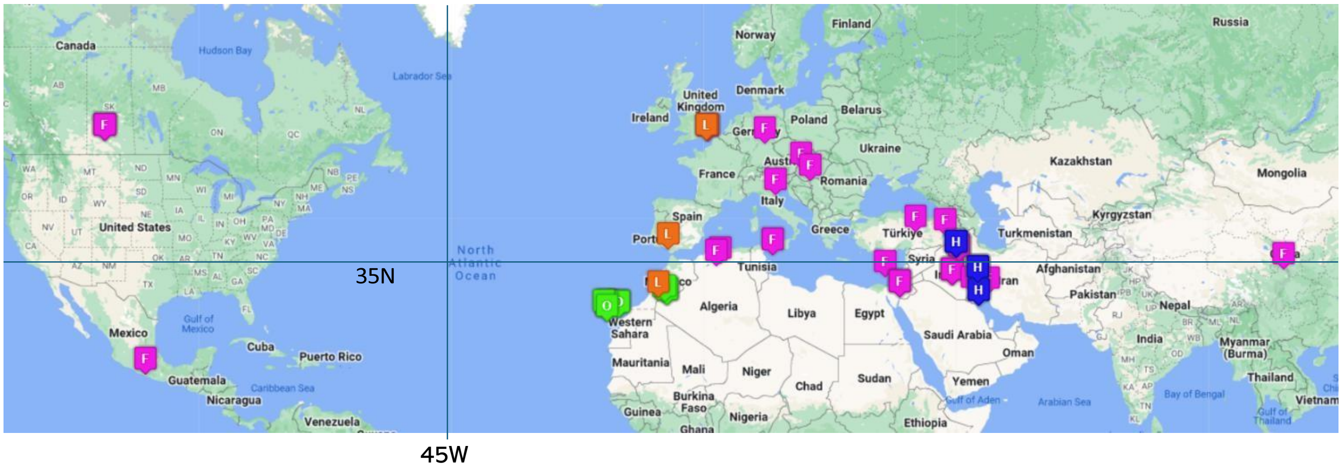

Supplementary Fig. 7. MDS and map of minor species. (a) Multi-dimensional scaling of n=9,111 taxa, highlighting only those accessions from species *A. fatua* (F, magenta), *A. hybrida* (H, blue), *A. occidentalis* (O, green) and *A. ludoviciana* (L, orange). Refer to Fig. 1b for comparison. The red circle shows the position of most A. *sativa*, which are hidden in this view. **(b)** Map of collection sites (when available) for taxa from the above plot. Source data are provided as a Source Data file.

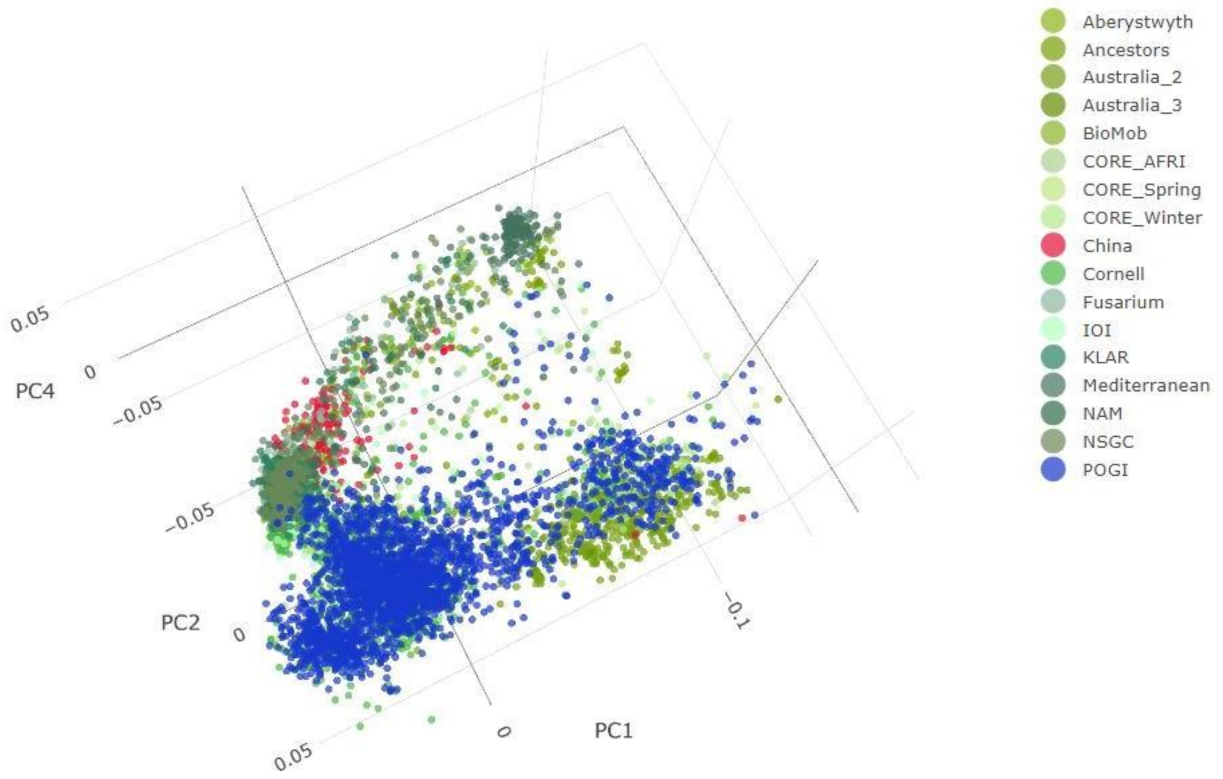

Supplementary Fig. 8. MDS showing Chinese taxa. Multi-dimensional scaling of n=6,950 taxa from populations containing primarily cultivated *A. sativa*. Accessions from China are highlighted in red, while those from POGI (A large North American study) are in blue. Online interactive versions of these plots can be used to visualize or highlight additional populations or features: (https://graingenes.shinyapps.io/Oat_diversity/). Source data are provided as a Source Data file.

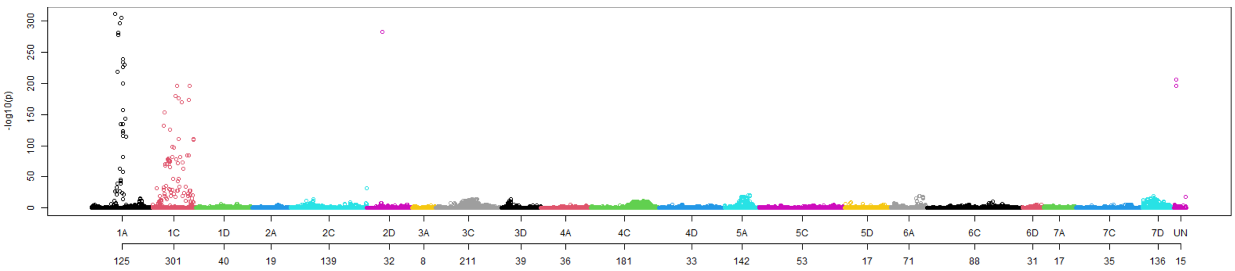

Supplementary Fig. 9. PCAdapt analysis. PCA-based genome-wide scans of local adaptation using PCAdapt analysis of the full set of taxa (*n*=8,816). The *Y*-axis shows the -log10(p) of the association of markers to population structure. The x-axis shows the chromosome names, and the secondary *x*-axis labels show the number of outlier loci associated with population structure below a threshold false discovery rate of 5%. Source data are provided as a Source Data file.

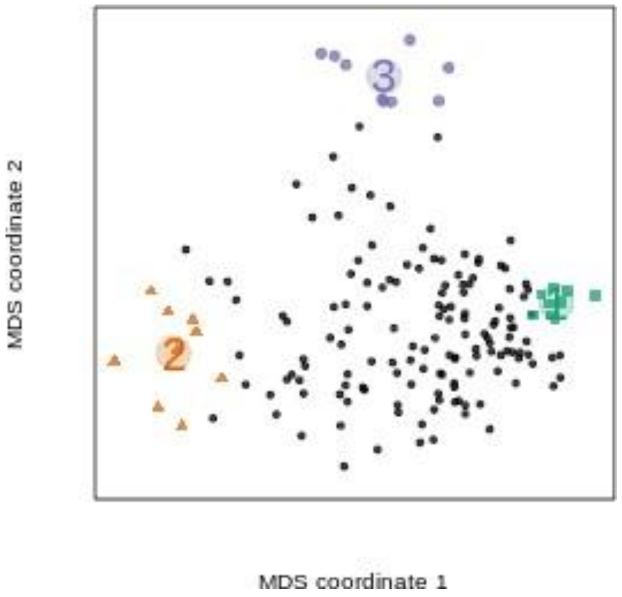

Supplementary Fig. 10. Lostruct analysis. Local structure analysis identified 27 outlier windows at three corners of the left multidimensional scaling plot. The green squares, orange triangles, and purple dots represent the genomic regions with abnormal population structure due to putative chromosomal inversions. Source data are provided as a Source Data file.

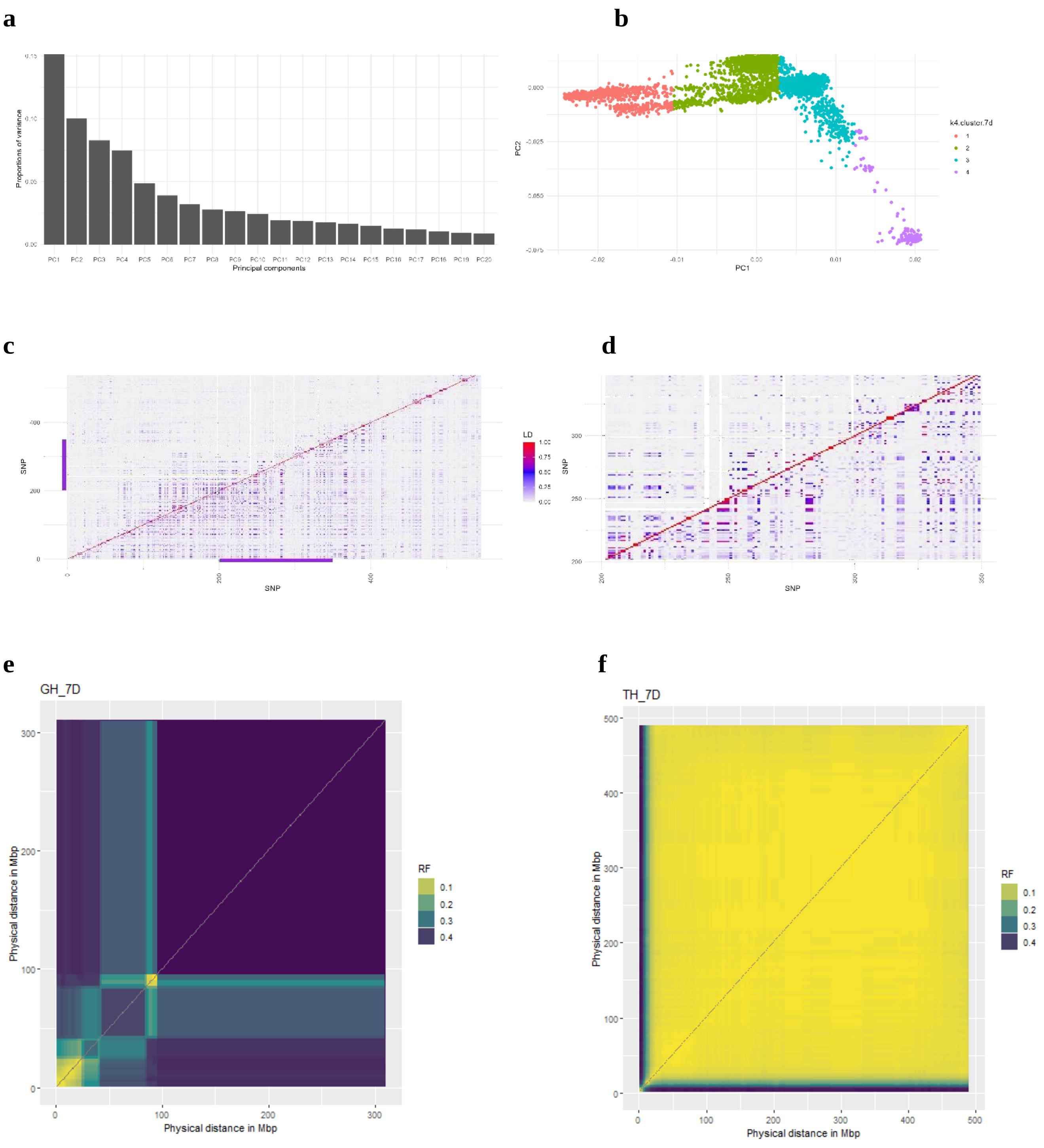

Supplementary Fig. 11. Detailed analysis of Chromosome 7D. A chromosomal inversion on chromosome 7D affects linkage and recombination patterns in oat. **(a)** The variance explained by the first 32 eigenvectors of the PCA analysis based on 150 SNPs in a 146 Mbp outlier region detected on chromosome 7D. **(b)** The distribution of 8,652 accessions along PC1 (*x*-axis) and PC2 (*y*-axis). The labels and colour of the circles from left to right show the *K*- means clustering (*K*=4, sum of squares between cluster/total 0.943) based on principal component one, from which we hypothesized the existence of four haplotypes: 7D-H1 (*n*=1,638), 7D-H2 (*n*=1,983), 7D-H3 (*n*=4,868), and 7D-H4 (*n*=163). **(c)** The LD on chromosome 7D of accessions classified as carrying 7D-H3 (the haplotype represented by the putative inversion carriers; *n*=4,868) above the diagonal and all 8,652 samples below the

diagonal. Purple bars show a putative 146 Mbp inversion region. **(d)** The LD within the putative inversion region of chromosome 7D (150 SNPs). **(e** and **f)**: Recombination frequency heatmaps of chromosome 7D of **(e)** progenies (*n*=160) from a cross between parental varieties ‘Goslin’ and ‘HiFi’ (both with haplotype 7D-H3) *vs*. **(f)** progenies (*n*=515) from a cross between TX07CS-1948 (haplotype 7D-H1) and ‘Hidalgo’ (haplotype 7D-H3)[8]. Source data are provided as a Source Data file.

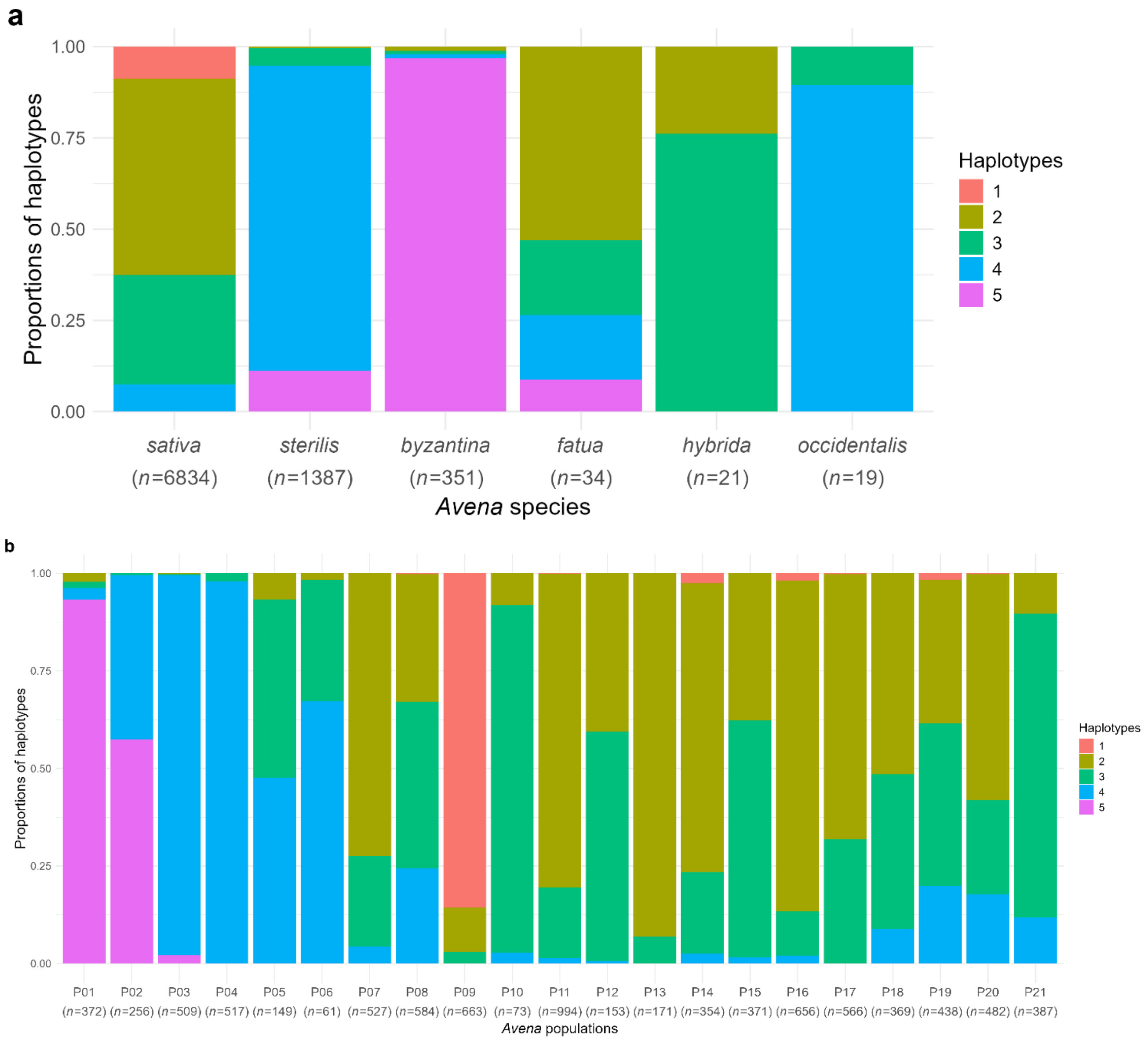

(a) **Supplementary Fig. 12. *In silico* karyotypes of the chromosome 1A.** The distribution of *in silico* karyotypes of the chromosome 1A translocation region in hexaploid *Avena* populations reveals signatures of domestication, population structure, breeding history, and agroecological adaptation. **(a)** The colours in the bar charts show the proportions of five haplotypes in each hexaploid species. **(b)** The proportion of the five haplotypes in each of the 21 *Avena* populations. The five haplotypes are identified in the legends by the final numeral in the following: 1AC-H1 (*n*=600), 1AC-H2 (*n*=3,706), 1AC-H3 (*n*=2,145), 1AC-H4 (*n*=1,695), and 1AC-H5 (*n*=506). The cross between 'Goslin' (having 1AC-H2) and 'HiFi' (having 1AC-H1) shows suppressed recombination and pseudo-linkage between 1A and 1C. In contrast, a cross between two parental lines which both have the translocated 1A/1C ('TX07CS-1948' (with 1AC-H3) x 'Hidalgo' (with 1AC-H2)) showed expected patterns of recombination (Kamal *et al*., Extended Data Fig. 6)[5], as did a cross between 'Terra' (with 1AC-H2) x 'Marion' (with 1AC-H2)[8]. Source data are provided as a Source Data file.

a

b

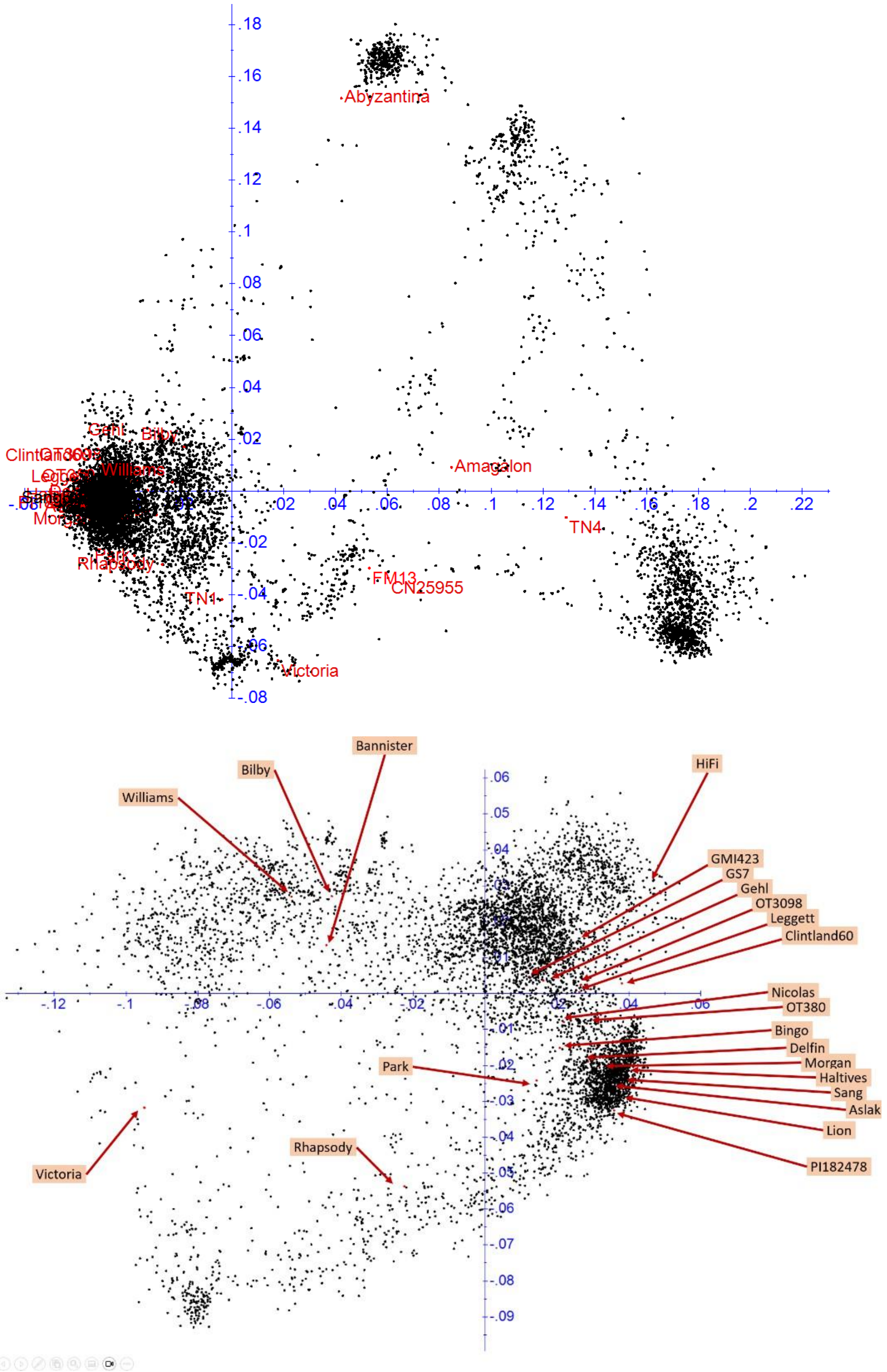

Supplementary Fig. 13. Location of reference genomes on MDS space. MDS analyses of **(a)** full data set (Matrix50) with the addition of reference genomes from the oat pan genome project[4]. **(b)** a reduced data set containing populations of *A. sativa* and the reference genomes that fall within this diversity space. Source data are provided as a Source Data file.

Supplementary references

1. Baum BR. *Oats: wild and cultivated. A monograph of the genus Avena L.(Poaceae)* (1977).
2. Diederichsen A. Assessments of genetic diversity within a world collection of cultivated hexaploid oat (*Avena sativa* L.) based on qualitative morphological characters. *Genetic Resources and Crop Evolution* **55**, 419-440 (2008).
3. Mascher M, Schreiber M, Scholz U, Graner A, Reif JC, Stein N. Genebank genomics bridges the gap between the conservation of crop diversity and plant breeding. *Nature Genetics* **51**, 1076-1081 (2019).
4. Avni R, *et al.* A pangenome and pantranscriptome of hexaploid oat. *bioRxiv*, https://www.biorxiv.org/content/10.1101/2024.10.23.619697v1 (2024).
5. Kamal N, *et al.* The mosaic oat genome gives insights into a uniquely healthy cereal crop. *Nature* **606**, 113-119 (2022).
6. Tinker NA, Bekele WA, Hattori J. Haplotag: software for haplotype-based genotyping-by-sequencing analysis. *G3: Genes Genomes Genetics* **6**, 857-863 (2016).
7. Bekele WA, Wight CP, Chao S, Howarth CJ, Tinker NA. Haplotype based genotyping-by-sequencing in oat genome research. *Plant Biotechnology Journal* **16**, 1452-1463 (2018).
8. Tinker NA, *et al.* Genome analysis in *Avena sativa* reveals hidden breeding barriers and opportunities for oat improvement. *Communications Biology* **5**, 474 (2022).
9. Lu F, *et al.* Switchgrass genomic diversity, ploidy, and evolution: novel insights from a network- based SNP discovery protocol. *PloS Genetics* **9**, e1003215 (2013).
10. Shen W, Le S, Li Y, Hu F. SeqKit: a cross-platform and ultrafast toolkit for FASTA/Q file manipulation. *PloS One* **11**, e0163962 (2016).
11. Camacho C, *et al.* BLAST+: architecture and applications. *BMC Bioinformatics* **10**, 1-9 (2009).
12. Bradbury PJ, Zhang Z, Kroon DE, Casstevens TM, Ramdoss Y, Buckler ES. TASSEL: software for association mapping of complex traits in diverse samples. *Bioinformatics* **23**, 2633-2635 (2007).
13. Perrier X, Jacquemoud-Collet JP. DARwin software http://darwin.cirad.fr/darwin.) (2006).
