# Supplementary material for: Global genomic population structure of wild and cultivated oat reveals signatures of chromosome rearrangements
Source: Nat Commun. 2025 Oct 29;16:9486. doi: 10.1038/s41467-025-57895-3 (PMC12572283; doi:10.1038/s41467-025-57895-3)
Supplement: Supplementary file 3 — Description of Additional Supplementary Files [file 41467_2025_57895_MOESM3_ESM.pdf]

Description of Additional Supplementary Files

File Name: Supplementary Data 1
Description: This file contains metadata for all accessions (Taxa) analyzed in the manuscript "Global genomic diversity analysis of wild and cultivated oat reveals population structure and signatures of chromosome rearrangements"

File Name: Supplementary Data 2
Description: Cross-referenced SNP calls: Reference-based vs Haplotag, indexed to the Sang (V1) Genome

File Name: Supplementary Data 3
Description: Taxa duplicates

File Name: Supplementary Data 4
Description: Phenotypes of genebank accessions.

File Name: Supplementary Data 5
Description: Barcodes and GBS information
