# Supplementary material for: Global genomic population structure of wild and cultivated oat reveals signatures of chromosome rearrangements
Source: Nat Commun. 2025 Oct 29;16:9486. doi: 10.1038/s41467-025-57895-3 (PMC12572283; doi:10.1038/s41467-025-57895-3)
Supplement: Supplementary file 9 — Reporting Summary [file 41467_2025_57895_MOESM9_ESM.pdf]

nature portfolio

Corresponding author(s): Wubishet Bekele, Nicholas Tinker, and Martin Mascher

Last updated by author(s): Feb 27, 2025

Reporting Summary

Nature Portfolio wishes to improve the reproducibility of the work that we publish. This form provides structure for consistency and transparency in reporting. For further information on Nature Portfolio policies, see our Editorial Policies and the Editorial Policy Checklist.

Statistics

For all statistical analyses, confirm that the following items are present in the figure legend, table legend, main text, or Methods section.

n/a	Confirmed	
☐	☒	The exact sample size (*n*) for each experimental group/condition, given as a discrete number and unit of measurement
☐	☒	A statement on whether measurements were taken from distinct samples or whether the same sample was measured repeatedly
☐	☒	The statistical test(s) used AND whether they are one- or two-sided<br>*Only common tests should be described solely by name; describe more complex techniques in the Methods section.*
☒	☐	A description of all covariates tested
☒	☐	A description of any assumptions or corrections, such as tests of normality and adjustment for multiple comparisons
☒	☐	A full description of the statistical parameters including central tendency (e.g. means) or other basic estimates (e.g. regression coefficient) AND variation (e.g. standard deviation) or associated estimates of uncertainty (e.g. confidence intervals)
☐	☒	For null hypothesis testing, the test statistic (e.g. *F*, *t*, *r*) with confidence intervals, effect sizes, degrees of freedom and *P* value noted<br>*Give P values as exact values whenever suitable.*
☒	☐	For Bayesian analysis, information on the choice of priors and Markov chain Monte Carlo settings
☒	☐	For hierarchical and complex designs, identification of the appropriate level for tests and full reporting of outcomes
☒	☐	Estimates of effect sizes (e.g. Cohen's *d*, Pearson's *r*), indicating how they were calculated

Our web collection on statistics for biologists contains articles on many of the points above.

Software and code

Policy information about availability of computer code

Data collection	No specialized software was used for data collection.
Data analysis	We used established R packages and bioinformatics software, all detailed in the Methods section, including the specific version of R used for most of the analysis. Sample scripts for each of 5 primary analyses are provided in Supplementary Data S7. Custom code was written to perform an analysis in Supplementary Fig. S13 and we have make this code and sample files available on Zenodo as cited under "Code Availability". Here are the list of software/packages and versions:<br>bwa 0.7.17-r1188<br>bcftools 1.9<br>picard 2.23.3<br>PLINK v1.90b6.21<br>R >4.3.2<br>stats 4.4.2 (Kmeans clustering, Mantel test)<br>TASSEL 5.2.94<br>SNPRelate 1.20.1<br>lostruct 0.0.0.9000<br>samtools 0.1.19<br>adegenet 2.1.10<br>ape 5.7-1<br>geosphere 1.5-18<br>LEA(sNMF) 3.12.2

vcfR 1.14.0
pcadapt 4.4.0
qvalue (FDR analysis) 2.36.0

For manuscripts utilizing custom algorithms or software that are central to the research but not yet described in published literature, software must be made available to editors and reviewers. We strongly encourage code deposition in a community repository (e.g. GitHub). See the Nature Portfolio guidelines for submitting code & software for further information.

Data

Policy information about availability of data

All manuscripts must include a data availability statement. This statement should provide the following information, where applicable:

- Accession codes, unique identifiers, or web links for publicly available datasets
- A description of any restrictions on data availability
- For clinical datasets or third party data, please ensure that the statement adheres to our policy

A full Data Availability statement is provided.

Research involving human participants, their data, or biological material

Policy information about studies with human participants or human data. See also policy information about sex, gender (identity/presentation), and sexual orientation and race, ethnicity and racism.

Reporting on sex and gender	*Use the terms sex (biological attribute) and gender (shaped by social and cultural circumstances) carefully in order to avoid confusing both terms. Indicate if findings apply to only one sex or gender; describe whether sex and gender were considered in study design; whether sex and/or gender was determined based on self-reporting or assigned and methods used.*<br>*Provide in the source data disaggregated sex and gender data, where this information has been collected, and if consent has been obtained for sharing of individual-level data; provide overall numbers in this Reporting Summary. Please state if this information has not been collected.*<br>*Report sex- and gender-based analyses where performed, justify reasons for lack of sex- and gender-based analysis.*
Reporting on race, ethnicity, or other socially relevant groupings	*Please specify the socially constructed or socially relevant categorization variable(s) used in your manuscript and explain why they were used. Please note that such variables should not be used as proxies for other socially constructed/relevant variables (for example, race or ethnicity should not be used as a proxy for socioeconomic status).*<br>*Provide clear definitions of the relevant terms used, how they were provided (by the participants/respondents, the researchers, or third parties), and the method(s) used to classify people into the different categories (e.g. self-report, census or administrative data, social media data, etc.)*<br>*Please provide details about how you controlled for confounding variables in your analyses.*
Population characteristics	*Describe the covariate-relevant population characteristics of the human research participants (e.g. age, genotypic information, past and current diagnosis and treatment categories). If you filled out the behavioural & social sciences study design questions and have nothing to add here, write "See above."*
Recruitment	*Describe how participants were recruited. Outline any potential self-selection bias or other biases that may be present and how these are likely to impact results.*
Ethics oversight	*Identify the organization(s) that approved the study protocol.*

Note that full information on the approval of the study protocol must also be provided in the manuscript.

Field-specific reporting

Please select the one below that is the best fit for your research. If you are not sure, read the appropriate sections before making your selection.

☒ Life sciences ☐ Behavioural & social sciences ☐ Ecological, evolutionary & environmental sciences

For a reference copy of the document with all sections, see nature.com/documents/nr-reporting-summary-flat.pdf

Life sciences study design

All studies must disclose on these points even when the disclosure is negative.

Sample size	This is a survey study of 9,153 taxa (accessions) of oat from diverse sources. Samples size as it applies to sequencing depth is indicated in the sample metadata. Sample size of the derived populations is indicated in all figures and secondary analyses.
Data exclusions	A total of 275 lines with low sequence coverage were excluded from the population structure analysis. Additionally, samples with more than 50% missing loci after imputation were excluded from the Lostruct analysis. Details of these data exclusions are provided in the Methods section.
Replication	No replicates were genotyped in this study. However, results for duplicated gene bank and breeding materials were analyzed and included in

Supplementary Analysis S1

Randomization	Randomization was not applicable to our study, as the data were compiled from published and unpublished sources.
Blinding	No deliberate blinding was applied in this study, but laboratories carrying out GBS and sequencing operations were not aware of sample identities.

Reporting for specific materials, systems and methods

We require information from authors about some types of materials, experimental systems and methods used in many studies. Here, indicate whether each material, system or method listed is relevant to your study. If you are not sure if a list item applies to your research, read the appropriate section before selecting a response.

Materials & experimental systems

n/a	Involved in the study	
☒	☐	Antibodies
☒	☐	Eukaryotic cell lines
☒	☐	Palaeontology and archaeology
☒	☐	Animals and other organisms
☒	☐	Clinical data
☒	☐	Dual use research of concern
☐	☒	Plants

Methods

n/a	Involved in the study	
☒	☐	ChIP-seq
☒	☐	Flow cytometry
☒	☐	MRI-based neuroimaging

Plants

Seed stocks	The sources of genotype data and accession information provided by the respective research groups or from publication records are summarized in the manuscript. This information will be hosted on a project website and updated regularly based on new data and feedback. Gene bank accession passport information is included for samples obtained from Plant Gene Resources of Canada (AAFC), as well as from the U.S. National Small Grains Collection (USDA-ARS). Seed stocks can be requested directly from these gene banks and research institutions.
Novel plant genotypes	NA
Authentication	NA
